# Correlation between Testicular Morphometric Parameters and Sperm Reserves in Ghanaian West African Dwarf Rams

**DOI:** 10.1155/2022/8421334

**Published:** 2022-04-14

**Authors:** Joseph Atawalna, Daniel Cobbinah Junior Essel, Nicholas Nettey, Patrick Mensah Amponsah

**Affiliations:** ^1^School of Veterinary Medicine, Kwame Nkrumah University of Science and Technology, Kumasi, Ghana; ^2^Regional Veterinary Laboratory, Veterinary Services Department, Kumasi, Ghana

## Abstract

The current study was conducted to investigate the association between testicular morphometric parameters and sperm reserves in Ghanaian West African Dwarf rams. The intact scrotum along with testes and epididymides from twelve rams were collected immediately after slaughtering and transported to the Regional Veterinary Laboratory. The left testes and epididymides from all rams were dissected and the testes were individually weighed. The individual length, width, and volume of the testis and cauda epididymis were determined. Tissue samples were taken from testicular parenchyma, homogenized, and used to determine gonadal sperm concentration using a hemocytometer. Spermatozoa were recovered from the cauda epididymis by the incision method and used to determine the spermiogram. The weight, length, width, and volume of the testis were 63 ± 11 gram, 6.7 ± 0.44 cm, and 4.6 ± 0.40 and 74 ± 19 cm^3^, respectively, while the same parameters (except weight) for the cauda epididymis were 3.2 ± 0.35 cm, 1.8 ± 0.16 cm, and 5.48 ± 1.5 cm^3^, respectively. The sperm reserves per testis (SR), sperm reserves per gram testis (SRG), daily sperm production per testis (DSP), and daily sperm production per gram testis (DSPG) were (11,740 ± 386, 250 ± 6, 2350 ± 7.7, and 50 ± 1.0) million sperm cells, respectively. The cauda epididymal sperm reserves were 1,386 ± 456 million sperm cells, and the sperm motility (M%), morphologically normal viable cells (N%), dead sperm cells (D%), and morphologically abnormal sperm cells (A%) were 89 ± 3.2, 70.13 ± 8.0, 13.55 ± 5.05, and 16.33 ± 7.9, respectively. In conclusion, testicular morphometric parameters in Ghanaian West African Dwarf rams are highly and positively correlated (*r* = 0.7487–0.9354) with sperm reserves and production.

## 1. Introduction

Small ruminant production plays an important role in the economies of developing countries. The major breeds of sheep reared in Ghana include the West African Draft (WAD) or Djallonke, long-legged Sahelian, and crossbreeds. The sheep population is estimated to be 4,335,000 [1]. Indigenous breeds are known to be more tolerant to existing livestock disease conditions and have higher growth and reproductive performance [2]. These factors make it desirable to maintain the genetic potential of indigenous breeds for sustainable livestock development. Artificial insemination and its associated techniques of semen collection, processing, and storage are very vital in this respect. Successful implementation of artificial insemination for sheep production will depend on the use of efficient breeding stock. In Ghana, some livestock breeding stations have been established and mandated to improve the performance of the native breeds of animals [3]. On these farms, male breeding parents are selected based on body weight and testicular morphometric parameters, which are good indicators of spermatozoa production [4]. Morphometric parameters are an essential component of breed characterization [5]. The assessment of potential breeding males should include testicular morphometry and estimation of sperm reserves and sperm quality parameters [6].

Evaluation of sperm reserves includes the assessment of gonadal and extragonadal sperm reserves, which are crucial in the selection of potential good breeders [7]. Extragonadal sperm reserves have been reported to be correlated with gonadal sperm reserves, testicular weight, and scrotal circumferences [8].

Sperm reserves have been evaluated from slaughtered domestic animals [9], while sperm quality parameters have been evaluated from sperm recovered from the cauda epididymis of recently slaughtered animals [10, 11]. The recovery of spermatozoa from the epididymides of recently dead or slaughtered valuable animals ensures the preservation of genetic material for future use [12]. Scarce information is available on the correlation of testicular morphometric parameters with sperm reserves, particularly in West African Dwarf rams in Ghana. Therefore, the current study was planned to investigate the correlation of testicular morphometric parameters with sperm reserves in the indigenous Ghanaian West African Dwarf rams.

## 2. Materials and Methods

### 2.1. Study Area

The study was conducted from May to June 2021, at the Akwatia Line slaughter slab, located in the centre of the Kumasi Metropolis. Kumasi is the regional capital of the Ashanti region and is located geographically within the forest zone of Ghana. It is a commercial city, where most animals brought for slaughtering, originate from Northern Ghana.

### 2.2. Sample Collection and Procession

The scrotal sacs from twelve clinically healthy WAD rams, aged 18–24 months, were collected immediately after slaughtering. The testes were collected after slaughtering because this procedure was less expensive. Moreover, the rams were humanely slaughtered at an approved slaughter facility where slaughtering processes adhered to laid down animal welfare principles. The procedures used in this study were approved by the animal ethics committee of the university. The body weight and age of the selected rams were estimated before slaughtering. The collected samples were packaged aseptically in polythene bags and transported on an ice chest to the Regional Veterinary Laboratory for analysis, within one hour after slaughtering. The left testes and epididymides were dissected, freed from all connective tissue and fats, and the testes were weighed individually with an electronic weighing scale (HRB series, HR1212004a, China). The length and width of the left testis and cauda epididymis in each sample were measured using Vernier calipers, while their respective volumes were estimated using the modified formula of a prolate spheroid [13] as follows: V = 0.5236 x L x W x (W)_2_, where L represents the length and W is the width of the testis.

### 2.3. Gonadal Sperm Reserve Estimation

Segments of testicular parenchyma were taken from the proximal and distal regions, weighed, and then homogenized with a high-speed blender for 2 min with 20 ml normal saline solution containing antibiotics (sodium penicillin G, 100 IU/mL, and streptomycin sulfate, 1 mg/mL) to prevent bacterial growth. The homogenate was stored overnight at 5°C to allow sperm cells to ooze out of the tissues. An aliquot of this homogenate was filtered and used to determine the gonadal sperm concentration, using an improved Neubauer hemocytometer after appropriate dilution in 0.05% formal saline as previously described [14]. The sperm reserves were calculated by multiplying the sperm concentration by the testicular volume. The daily sperm production per testis was obtained by dividing the gonadal sperm reserves by a time divisor of 5 days, corresponding to the duration of the period of the seminiferous epithelium cycle when spermatids are resistant to homogenisation in rams [15].

### 2.4. Cauda Epididymal Sperm Quality

Spermatozoa were collected from the cauda epididymis by multiple longitudinal incisions into its parenchyma, followed by immersion into a Petri dish containing 2.9% sodium citrate buffer solution and placed in a water bath at 37°C for 30 minutes [16]. Cauda epididymal sperm concentration was measured using an improved Neubauer hemocytometer [14]. Sperm motility was measured by mixing a drop of semen and a drop of 2.9% sodium citrate buffer on a prewarmed slide and observing under a microscope at 400x magnification. Sperm morphology was determined using the Nigrosin-eosin stain [17].

### 2.5. Data Analysis

The general linear model was used to analyse the results. The results obtained were expressed in the form of mean ± SD, using the GraphPad Prism software [18]. The association between morphometry and sperm production was determined using Pearson's coefficient of correlation (*r*).

## 3. Results

### 3.1. Testicular Morphometry

The data for morphometric parameters of the testes and cauda epididymides of WAD rams are presented in Table 1. The mean values of body weight of rams, weight, length, width, and volume of the testes were 28 ± 5.1 Kg, 63 ± 11 gm, 6.7 ± 0.44 cm, and 4.6 ± 0.40 and 74 ± 19 cm^3^, respectively, while the length, width, and volume of the cauda epididymides were 3.2 ± 0.35 cm, 1.8 ± 0.16 cm, and 5.48 ± 1.5 cm^3^, respectively.

### 3.2. Gonadal Sperm Reserves

The mean values of sperm reserve of whole testis (SR), sperm reserve per gram testis (SRG), daily sperm production per testis (DSP), and daily sperm production per gram testis (DSPG) were 11,740 ± 386, 250 ± 6, 2350 ± 7.7, and 50 ± 1.0 million sperm cells, respectively (Table 2).

### 3.3. Correlation between Testicular Morphometric Parameters and Sperm Production

The correlations between testicular morphometric parameters and sperm production are illustrated in Table 3. The body weight of rams correlated highly and positively (*r* = 0.8262–0.9676) with the weight, length, width, and volume of testes and also with sperm reserves and sperm production. Testicular morphometric parameters correlated highly and positively with each other (*r* = 0.7487–0.9354) and also with sperm reserves and sperm production. Sperm reserves correlated positively and highly (*r* = 0.9804–1.000) with sperm reserves per gram testis, daily sperm production, and daily sperm production per gram testis.

### 3.4. Cauda Epididymal Sperms

The cauda epididymal sperm reserves were 1,386 ± 456 million sperm cells, while the sperm motility (M%), morphologically normal viable cells (N%), dead sperm cells (D%), and morphologically abnormal sperm cells (A%) were 89 ± 3.2, 70.13 ± 8.0, 13.55 ± 5.05, and 16.33 ± 7.9, respectively (Table 4).

## 4. Discussion

### 4.1. Testicular Morphometry

Our results in terms of length and width of the testis were similar to the values of 6.3 ± 0.16 cm and 4.4 ± 0.11 cm, respectively, as previously documented in WAD rams in North Benin [19]. However, the rams enrolled in the current study were heavier than those in Benin.

The mean values of weight and volume of the testis of rams in the current study were higher than 51.20 gm and 53.7 cm^3^, respectively, reported for WAD rams in Akwa Ibom State, Nigeria [20]. These differences may be due to variation in geographical location and age (younger rams of 6–9 months of age in the former study). Although the referred study did not consider the association between live body weight and testicular morphometry, other research concluded that testicular morphometric parameters were strongly and positively associated with live weight [19, 20]. The WAD sheep breed has been reported to be smaller in size compared to other West African breeds such as Ouda, Balami, and Yankasa [21].

However, the mean values of testes' weight, length of testes, and cauda epididymides were lower than 99.8 ± 2.6 gram, 8.7 ± 0.2 cm, and 4.4 ± 0.7 cm, respectively, as reported for Yankasa rams reared in Zaria, Nigeria [22]. Moreover, Yankasa rams are usually larger than WAD rams and have comparatively higher testicular length, testicular width, and volume [13].

### 4.2. Gonadal Sperm Reserves

The mean values of SR, DSP, and DSPG in this study were higher than 2,150 ± 110, 600 ± 10, and 9.4 ± 2 million sperm cells, respectively, reported for WAD rams in Nigeria [23]. These differences may be due to the variations in geographical location and nutritional levels of the rams. The DSPG of WAD rams in the current study was higher than 36 ± 14.6 million sperm cells reported for Yankassa rams in Nigeria [22]. Similarly, the gonadal sperm reserves and cauda epididymal sperm reserves of the WAD rams in the study were higher than 112.5 ± 21.77 and 1,378.50 ± 60.80 million sperm cells, respectively, as compared to Yankasa rams in Nigeria [22]. These findings suggest that WAD rams have higher spermatogenic action compared to Yankasa rams.

### 4.3. Correlation between Testicular Morphometric Parameters and Sperm Reserves

The testicular morphometric parameters highly correlated with each other and with sperm reserves and daily sperm production. Our findings agree with previous reports that high correlations existed between testicular weight, sperm reserves, and daily sperm production in WAD rams [23]. Moreover, in Murrah bulls, there were high correlations between scrotal circumference, testicular length, testicular diameter, testicular volume, and body weight [24, 25]. Similarly, testicular length correlated positively and moderately with testicular sperm reserves in Jordanian native bucks [11], while testicular morphometric parameters correlated highly with each other and with epididymal sperm reserves in Nigerian Sahel goats [9].

### 4.4. Cauda Epididymal Sperm

The mean values of cauda epididymal sperm reserves in this study were higher than reported for WAD rams in Ibadan, Nigeria [26], and Yankasa rams in Nigeria [22]. On the contrary, these values were lower than 20.37 ± 0.77 billion sperm cells, documented for cauda epididymal sperm reserves in the local breed of sheep in Kashmir, India [27]. The differences in sperm reserves may be due to variation in breed, body weight, and weight of epididymides.

The mean values of percent sperm motility, sperm viability, and morphological abnormal sperms in this study were within the ranges of 66.25–85.66, 84.17–87.66, and 11.84–12.96, respectively, as documented for WAD rams in Ibadan, Nigeria [28]. On the contrary, the percent sperm motility and viability were lower than 83.33 ± 1.05 and 93.85 ± 1.98, respectively, for native rams in India [29]. These differences may be due to variations in breeds and geographical location.

## 5. Conclusion

In conclusion, testicular morphometric parameters in Ghanaian West African Dwarf rams were highly and positively correlated (*r* = 0.7487–0.9354) with sperm reserves and production. Future research should be conducted to investigate the fertilizing ability of spermatozoa recovered from recently slaughtered animals.

## Figures and Tables

**Table 1:** Gross testicular morphometric parameters in Ghanaian WAD rams (*n* = 12).

Parameter	Mean ± SD
Body weight/Kg	28 ± 5.1
Testis weight/gm	63 ± 11
Total testis weight/gm	124 ± 23.04
Testis length/cm	6.7 ± 0.44
Testis width/cm	4.6 ± 0.40
Testis volume/ml	74 ± 19
Cauda epididymis length/cm	3.2 ± 0.35
Cauda epididymis width/cm	1.8 ± 0.16
Cauda epididymis volume/cm^3^	5.48 ± 1.468
Gonadosomatic index (GSI) (%)	0.44 ± 0.039

**Table 2:** Gonadal sperm reserves in Ghanaian WAD rams (*n* = 12).

Parameter/(×10^6^)ml	Mean ± SD
Sperm reserves per testis	11.740 ± 390
Sperm reserves per gram testis	250 ± 6
Daily sperm production	2.350 ± 77
Daily sperm production per gram testis	50 ± 1

**Table 3:** Coefficient (*r*) of correlation between testicular morphometric parameters, sperm reserves, and sperm production in Ghanaian WAD rams (*n* = 12).

	DSPG	DSP	SRG	SR	VT	WdT	LT	WT
WT	0.8262	0.8740	0.8262	0.8740	0.9676	0.9525	0.967	—
LT	0.7487	0.7975	0.7487	0.7975	0.9354	0.8964	—	
WdT	0.8318	0.9048	0.8318	0.9048	0.9907	—		
VT	0.8227	0.8940	0.8227	0.8940	—			
SR	0.9864	1.000	0.9864	—				
SRG	1.000	0.9874	—					
DSP	0.9864	—						
DSPG	—							

WT, body weight; LTW, left testicular weight; LTL, left testicular length; WdLT, width of left testes; LTV, left testicular volume; SR, sperm reserves per testis; SRG, sperm reserve per gram testes; DSP, daily sperm production; DSPG, daily sperm production per gram testes; r, Pearson coefficient of correlation.

**Table 4:** Cauda epididymal sperm parameters.

Parameter	Mean ± SD
Cauda epididymal sperm reserves (10^6^)	1.386 ± 456
Sperm motility (M) (%)	89 ± 3.2
Morphologically normal sperms (N) (%)	70.13 ± 8.0
Dead sperms (D) (%)	13.55 ± 5.05
Morphologically abnormal sperms (A) (%)	16.33 ± 7.9

## Data Availability

The data used to support the research findings are included within the article.
